# Genome-Wide Identification and Functional Analysis of the bZIP Transcription Factor Family in Rice Bakanae Disease Pathogen, *Fusarium fujikuroi*

**DOI:** 10.3390/ijms23126658

**Published:** 2022-06-15

**Authors:** Kehan Zhao, Lianmeng Liu, Shiwen Huang

**Affiliations:** China National Rice Research Institute, Hangzhou 310006, China; khanzhao0202@163.com

**Keywords:** bZIP TF, bakanae disease, *Fusarium fujikuroi*, phylogenetic analysis, functional analysis

## Abstract

Fungal basic leucine zipper (bZIP) proteins play a vital role in biological processes such as growth, biotic/abiotic stress responses, nutrient utilization, and invasion. In this study, genome-wide identification of bZIP genes in the fungus *Fusarium fujikuroi*, the pathogen of bakanae disease, was carried out. Forty-four genes encoding bZIP transcription factors (TFs) from the genome of *F. fujikuroi* (*FfbZIP*) were identified and functionally characterized. Structures, domains, and phylogenetic relationships of the sequences were analyzed by bioinformatic approaches. Based on the phylogenetic relationships with the FfbZIP proteins of eight other fungi, the bZIP genes can be divided into six groups (A–F). The additional conserved motifs have been identified and their possible functions were predicted. To analyze functions of the bZIP genes, 11 *FfbZIPs* were selected according to different motifs they contained and were knocked out by genetic recombination. Results of the characteristic studies revealed that these *FfbZIP*s were involved in oxygen stress, osmotic stress, cell wall selection pressure, cellulose utilization, cell wall penetration, and pathogenicity. In conclusion, this study enhanced understandings of the evolution and regulatory mechanism of the *FfbZIPs* in fungal growth, abiotic/biotic stress resistance, and pathogenicity, which could be the reference for other fungal bZIP studies.

## 1. Introduction

Rice is one of the most important food crops in the world and more than 50% of the world population depends on rice as their staple food. The bakanae disease of rice what caused by *Fusarium fujikuroi* was one of a seed born disease and wide distribution in rice growing area in the world. In terms of the rice plant, bakanae disease causes slenderly growth, seedling and plant death, and substantial yield reduction, bringing about enormous economic loss and even threatening food security. According to the statistics, bakanae disease could cause a 0–10% yield reduction in a mild case, but up to 30–95% yield loss in a serious case [1,2,3,4].

Transcription factor (TF), also known as trans-acting factor, is a protein that regulates gene expression in eukaryotes. It participates in specifically binding to cis-acting elements in the upstream promoter sequence of structural genes and takes an important role in activating or inhibiting transcription [5]. TFs activate the activity of transcription complexes containing RNA polymerase II, thereby regulating the coordinated transcription and expression of multiple target structural genes and playing a crucial role in the life process of organisms [6]. So far, more than 90 types of TFs have been reported in eukaryotes [7]. In the whole genome of more than 200 fungi, over 80 typical TF types were annotated, in addition to basic region/leucine zipper motif (bZIP), including TFs common in fungi such as Zn(II)_2_Cys_6_ zinc cluster protein (C_6_ zinc fingers) [8], Cys_2_His_2_ zinc finger protein (C_2_H_2_ zinc fingers), basic Helix-Loop-Helix (bHLH), GATA protein, MADS-box protein, MYB-like protein [9,10], and and homeodomain protein.

bZIP TFs are one of the most widely distributed and conserved TFs of eukaryotes. bZIP proteins contain two domains—a highly conserved DNA-binding basic region and a diverse leucine zipper [11]. In the form of homodimers or heterodimers, bZIP TFs regulate downstream-related gene expression such as *ERF5*, *COR78*, *CYP707A3*, and *ADS* by binding to cis-acting elements G-box, C-box, ABRE, LTRE, and Box II [9]. bZIP TFs are located in the nucleus and participate in various biological processes [12], for example, YAP-type bZIP TFs respond to oxidative stress [13,14], Atf-type bZIP TFs respond to osmotic stress [15,16,17], and Hac-type bZIP TFs are associated with non-folded proteins [18,19].

Scholars have carried out a lot of research on bakanae disease pathogens, but the interaction mechanism between bakanae disease pathogens and rice is still unclear, the same as the related molecular regulatory mechanism. Lack of knowledge of the molecular mechanism of bakanae disease pathogens is not conducive to understanding the regulation of the pathogenicity of bakanae disease pathogens, as well as the screening and utilization of related resistance genes. In this study, we identified 44 bZIP TFs from *F. fujikuroi*, analyzed genome-wide systematic characterization, and performed phylogenetic analysis with eight other common fungal bZIP TFs to understand their phylogenetic relationships with each other. In order to elucidate the biological function of *FfbZIP* genes, 11 *FfbZIP* genes were selected according to different domains, and deletion mutants were obtained by gene knockout. Results of functional experiments indicated that FfbZIP TFs not only played a regulatory role in the process of fungal growth and development, nutrient absorption and utilization, and stress response, but also regulated the invasion process of *F. fujikuroi*. The genome-wide and functional analysis of FfbZIP TFs in this study contributed to the research on the molecular mechanism of pathogenicity and expanded the research field of fungal bZIP TFs.

## 2. Results

### 2.1. Identification of the FfbZIP Transcription Factor Family from F. fusarium

In the Pfam database, there are three conserved bZIP domain sequences with domain IDs PF00170 (bZIP_1 domain), PF07716 (bZIP_2), and PF03131 (bZIP_Maf). Based on this, an alignment search was performed on the *F. fujikuroi* bZIP TF family proteins using HMM, and 37 matches were obtained. 186 bZIP protein sequnces of *F. graminearum*, *F. oxysporum*, *F. verticillioides, F. solani*, *S. cerevisiae*, *U. virens*, *N. crassa*, and *M. oryzae* were used as templates to search through the F. fujikuroi genome with TBLASTN, and 57 matches were obtained. Repeated sequences were deleted, and the obtained protein sequences were screened by CD-Search. Some protein sequences without bZIP domain were eliminated. Finally, a total of 44 predicted *F. fujikuroi* bZIP TF protein sequences were obtained from the *F. fujikuroi* genome. These genes were designated as *FfbZIP1* to *FfbZIP44* according to their locus number ordering. The gene name, locus number, gene symbol, gene ID, chromosome position, genomic location, protein size and ORF size on supercontig of all 44 *FfbZIP*s are indicated in Table 1.

### 2.2. Conserved Domain and Structure of FfbZIP Protein

**Figure 1:**
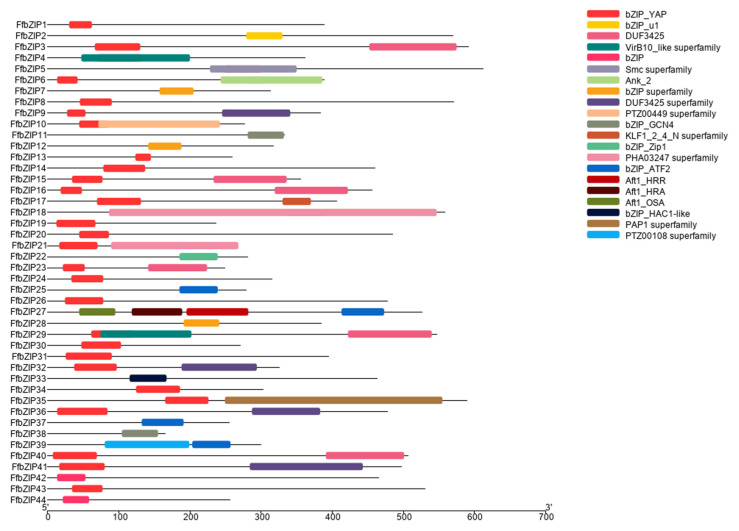
Schematic diagram of the conserved domains of FfbZIP proteins.

In the predicted FfbZIP TF protein sequences, in addition to 19 FfbZIP TFs with only one domain, the bZIP domain, 12 of these protein sequences also contained other predicted domains. FfbZIP3 contained VirB10_like domain, and FfbZIP4 contained Smc superfamily domain, and both domains overlapped with the bZIP domain. FfbZIP7 with PTZ00449 superfamily domain at the N-terminal of the protein, and the domain partial overlapped with the C-terminus of the bZIP domain. FfbZIP13 contained KLF1_2_4_N superfamily domain, FfbZIP22 contained PAP1 superfamily domain and FfbZIP24 contained PTZ00108 superfamily domain, all domains do not show overlap with bZIP domain. FfbZIP17 contained four kinds of Atf domains, including Atf1_HRR, Atf1_HRA, Atf1_OSA and bZIP_ATF2. In addition, FfbZIP11, FfbZIP12, FfbZIP23, FfbZIP27, and FfbZIP28 all contained DUF domains, which are DUF3425 and DUF3425 superfamily (Figure 1).

**Figure 2:**
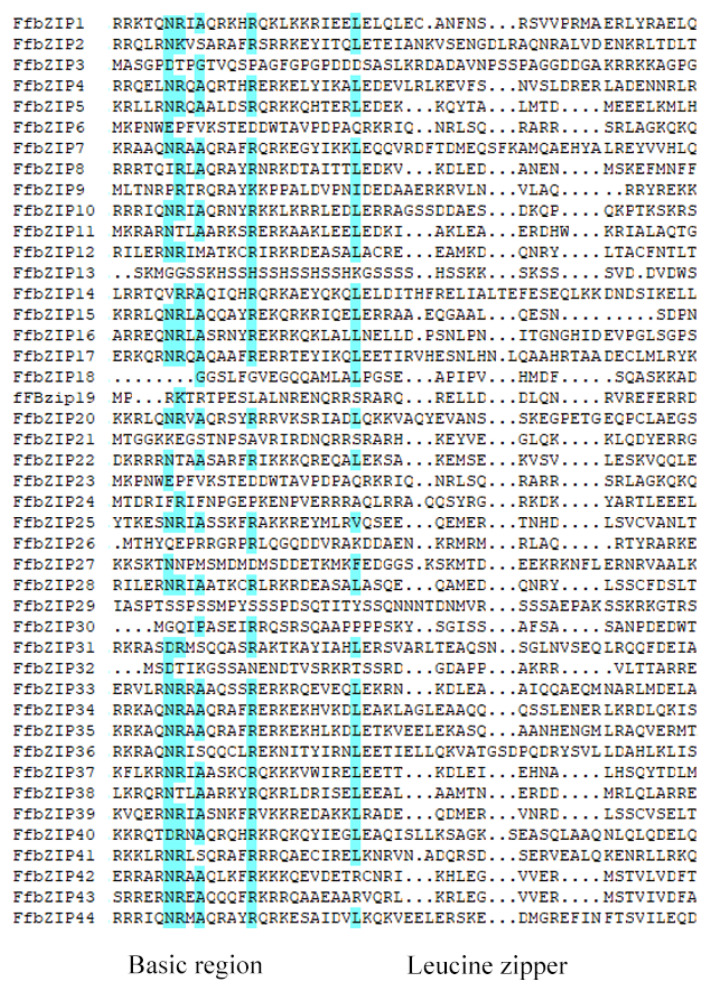
Sequence alignment of FfbZIP domains.

The N-terminal basic amino acid region of the bZIP domain was highly conserved, containing a nuclear localization signal (NLS) [20] and an N-x7-R/K domain consisting of 16–20 amino acid residues, among which the last amino acid is arginine (R) or lysine (K) [9]. The leucine zipper region was not conserved and consists of several leucine repeat heptapeptides or hydrophobic amino acid residues (such as Ile, Val, Phe or Met). The leucine zipper region involved in oligomerization was connected to the basic region. Leucine is at position seventh of every seven amino acids, and sometimes replaced by isoleucine, valine, phenylalanine, or methionine [21]. In order to study the characteristics of FfbZIP domains, the amino acid sequences were compared and analyzed. All basic regions of FfbZIP TFs contained an N-x7-R domain but not an N-x7-K domain, suggesting that arginine was more conserved than lysine in FfbZIP proteins. In the leucine zipper region, the leucine in the first repeat heptapeptide was highly conserved. Two leucines (L) were replaced by valine (V) or arginine (R). In the latter two repeat heptapeptides, there were a lot of variation (Figure 2).

### 2.3. Intron Distribution Analysis of FfbZIP Gene

**Figure 3:**
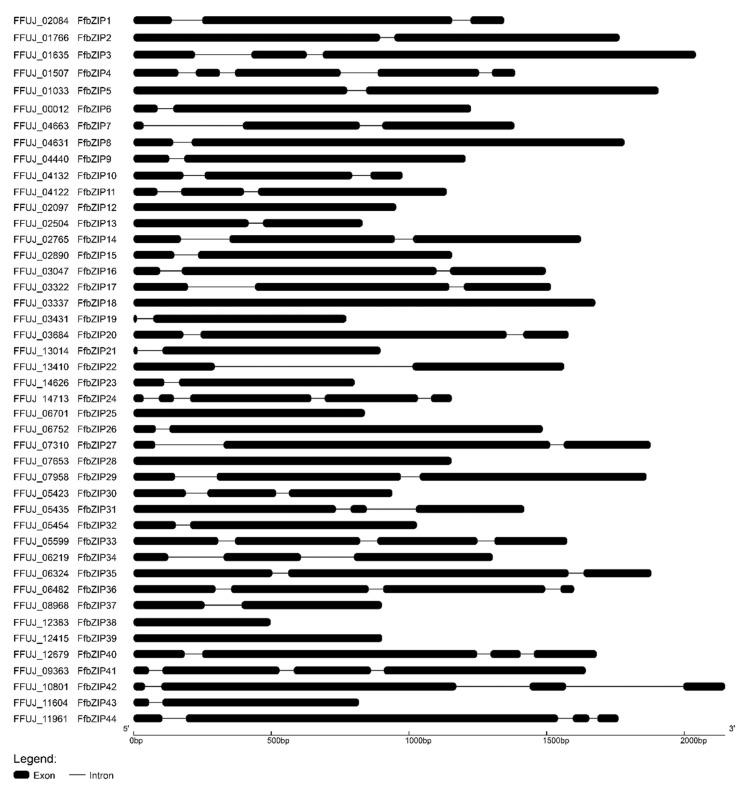
Intron and exon distribution patterns in the coding sequence of *FfbZIP* genes.

To gain insights into the structural evolution of the *FfbZIP* genes, the intron distribution in *FfbZIP* genes was subjected to alignment analysis using GSDS. As shown in Figure 3, all *FfbZIP* genes contained 1–4 introns except *FfbZIP12*, *FfbZIP18*, *FfbZIP25*, *FfbZIP28, FfbZIP38* and *FfbZIP39*. The *FfbZIP* genes were classified into five classes depending on their different intron numbers. Most *FfbZIP* genes contained two introns (34.09%, 15) and single-intron *FfbZIP* genes (34.09%, 15), followed by three introns (13.63%, 6), intronless (13.63%, 6), and four introns (4.56%, 2). There was no obvious relationship between exon length, intron length and intron number, neither the relationship between exon length and intron length (Supplementary Figure S1).

### 2.4. Phylogenetic Analysis of FfbZIP Proteins

To investigate the phylogenetic relationships of the *FfbZIP* genes and relationships with other fungal bZIP proteins, 230 bZIP proteins from *F. fujikuroi*, *F. graminearum*, *F. oxysporum*, *F. verticillioides, F. solani*, *S. cerevisiae*, *U. virens*, *N. crassa*, and *M. oryzae* were analyzed (Supplementary Figure S2). The bZIP proteins were divided into six clades, and designated as A to F, indicating that the evolution of these bZIP genes were conserved between the nine fungi. In clades B and C there were bZIP proteins from nine fungi, while clade A was missing the bZIP protein of *S. cerevisiae*, clade D and E were missing the bZIP protein of *N. crassa*, and clade F was missing the bZIP protein of *F. graminearum*, *F. verticillioides* and *N. crassa*. Evolutionary analysis also revealed a closer homology relationship between *F. fujikuroi* and *F. oxysporum* (nine bZIP proteins with high homology), five with *F. graminearum*, four with *F. verticillioides*, three with *U. virens*, two with *F. solani* and *M. oryzae*, and one with *S. cerevisiae*.

### 2.5. Construction and Identification of FfbZIP Deletion Mutants

Generally, each domain had a unique spatial conformation and undertook different biological functions. To explore the effect of different domains on the function of FfbZIP TFs, the deletion mutants of *FfbZIP* genes with different domains were constructed. FfbZIP TFs contained bZIP_u1, bZIP_YAP, VirB10, bZIP, Smc, PTZ00449, bZIP_GCN4, DUF3425, KLF1_2_4_N, bZIP_Zip1, PAP1 domains, and the corresponding genes were *FfbZIP2*, *FfbZIP4*, *FfbZIP5*, *FfbZIP8*, *FfbZIP10*, *FfbZIP11*, *FfbZIP16*, *FfbZIP17*, *FfbZIP22*, *FfbZIP35*, and *FfbZIP44*. To characterize the functional roles of *FfbZIP* genes on biological functions and pathogenicity, targeted deletion of these genes was achieved by homologous recombination.

F/R primers were used to detect the presence or absence of HygR or target gene at a specific position at the same time. Due to the difference in the length of the target gene and the hygromycin resistance gene, if the wild-type and transformant amplified fragments have different lengths and no identical bands, then it shows that the hygromycin resistance gene has replaced the target gene, that is, the target gene has been knocked out. Taking the construction of the Δ*FfbZIP10* mutant as an example, the hygromycin resistance gene fragment was inserted into the upstream and downstream 2000 bp gene sequences of the *FfbZIP10* gene using SnapGene software, and the NCBI Primer-BLAST website (https://www.ncbi.nlm.nih.gov/tools/primer-blast/index.cgi?LINK_LOC=BlastHome/, accessed on 5 June 2022) found two upstream primers within the upstream 500 bp range, named UbZIP10F1 and UbZIP10F2, respectively, and similarly found two downstream primers in the downstream sequence and named them as DbZIP10R2, DbZIP10R1, at the upstream junction with the hygromycin resistance gene, took 20 bp of gene sequence totaling 40 bp, set as primer UbZIP10R, and similarly obtained primer DbZIP10F at the junction between the downstream gene and the resistance gene. Two primers, H2 and H3, were obtained at the position of about three equal parts of the hygromycin resistance gene, the knockout fragment was obtained by PCR technology, and the transformant was obtained by PEG mediation. The inserted gene was stably inherited after three generations of continuous culture, and a total of 21 transformants were obtained. The transformants were detected by PCR using DbZIP10F/UbZIP10R primers. The amplification product of *FfbZIP10* is shorter than the amplification product of the resistance gene, and the band of the wild-type PCR amplification product was lower than that of the transformant, that is, T1, T2, T4 and T6 were positive transformants, and T3 and T5 indicated that the hygromycin resistance gene was inserted but not at the location of the *FfbZIP10* gene, but was inserted randomly, so the PCR amplification result was two bands (Supplementary Figure S3).

### 2.6. Phenotypic Observation of FfbZIP Deletion Mutants

#### 2.6.1. Fungi Morphology Observation

**Figure 4:**
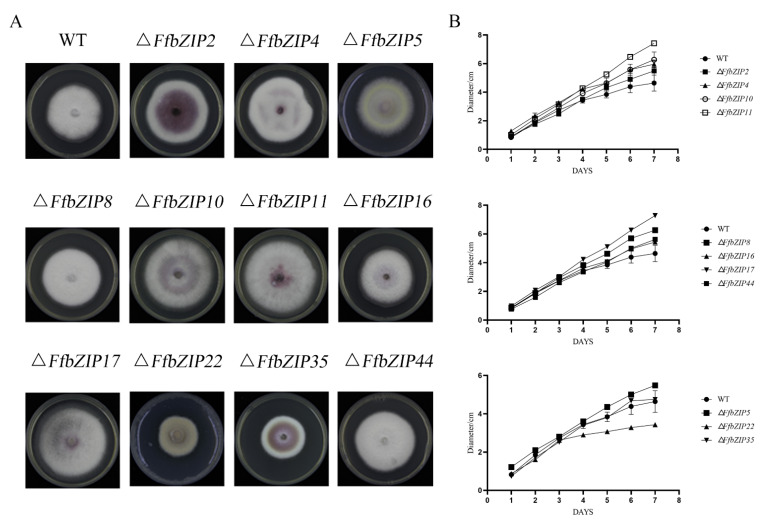
Fungi morphology observation. (**A**) Colony morphology of *FfbZIP* knockout mutant strain. (**B**) Growth rate of *FfbZIP* knockout mutants versus wild type. Error bars represent means ± SEs. Some error bars were not plotted because the error bar would be shorter than the size of the symbol.

The comparison of the strain morphology between *F. fujikuroi* wild-type and *FfbZIP* deletion mutants on PDA medium in Figure 4 showed that Δ*FfbZIP2*, Δ*FfbZIP4*, Δ*FfbZIP5*, Δ*FfbZIP16*, and Δ*FfbZIP35* were not significantly different from wild type at 7 days post inoculation (DPI). Δ*FfbZIP35* was smaller than wild type, and the growth rate was slower than wild type after 3 days’ culturing by observation. The strains of other deletion mutants were larger than the wild type, and the growth rates were faster after 2–4 days’ culturing. The results showed that *FfbZIP2*, *FfbZIP4*, *FfbZIP5*, *FfbZIP16*, and *FfbZIP35* had nothing to do with strain growth, *FfbZIP22* promoted strain growth, and the other *FfbZIP* genes inhibited strain growth of *F. fujikuroi*.

*F. fujikuroi* wild-type strains were white, and only a small amount of purple pigment could be observed at the bottom. Δ*FfbZIP2* and Δ*FfbZIP35* produced a large amount of purple pigment, while Δ*FfbZIP4*, Δ*FfbZIP10*, Δ*FfbZIP11* and Δ*FfbZIP17* produced slightly more purple pigment than the wild type, and the rest of the deletion mutants were no different from the wild type. The coloration phenotypes were maintained in the whole population of positive deletion mutants. The results revealed that *FfbZIP2* and *FfbZIP35* inhibited the synthesis of purple pigment, while *FfbZIP4*, *FfbZIP10*, *FfbZIP11*, and *FfbZIP17* inhibited to a certain extent, and other *FfbZIP* genes did not participate in the synthesis of purple pigment.

We discovered by observation that wild type produced a large number of aerial hyphae, while Δ*FfbZIP5* and Δ*FfbZIP22* were without it. The aerial hyphae of Δ*FfbZIP10* and Δ*FfbZIP17* were slightly less than those of the wild type, and the other deletion mutants were consistent with the wild type. The results indicated that *FfbZIP5* and *FfbZIP22* promoted the formation of aerial hyphae, *FfbZIP10* and *FfbZIP17* had positive regulatory effects on the formation of aerial hyphae, and the other *FfbZIP* genes did not affect the formation of aerial hyphae.

#### 2.6.2. Oxidative Stress Experiment

**Figure 5:**
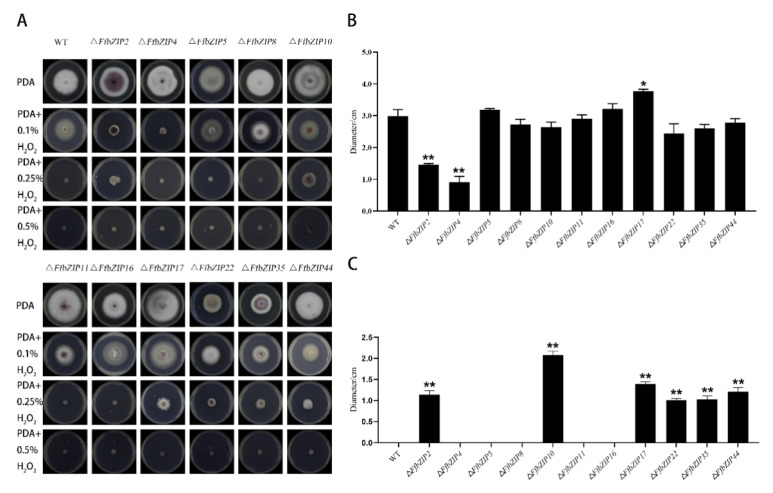
Oxidative stress experiment. (**A**) Growth of wild type and *FfbZIP* knockout mutants under exogenous oxygen stress. (**B**) Histogram of strain diameter under 0.1% H_2_O_2_ oxidative stress. Error bars represent means ± SEs. (**C**) Histogram of strain diameter under 0.25% H_2_O_2_ oxidative stress. Error bars represent means ± SEs. * *p* < 0.05, ** *p* < 0.01.

Δ*FfbZIP4* could not grow under 0.1% H_2_O_2_, while Δ*FfbZIP2* grew much smaller than the wild type (Figure 5). Δ*FfbZIP17* was significantly larger than wild type at *p* = 0.05 level, but not at *p* = 0.01 level. The other deletion mutants were not significantly different from the wild type. Under 0.25% H_2_O_2_, Δ*FfbZIP5*, Δ*FfbZIP8*, Δ*FfbZIP11*, and Δ*FfbZIP16* lost the growth ability, like the wild type. Δ*FfbZIP2*, Δ*FfbZIP10*, Δ*FfbZIP17*, Δ*FfbZIP22*, Δ*FfbZIP35* and Δ*FfbZIP44* grew to be tiny. Neither wild type nor deletion mutants could grow under 0.5% H_2_O_2_. The results showed that *FfbZIP2* and *FfbZIP4* positively regulated the anti-oxidative stress of *F. fujikuroi* under low oxidative stress conditions, while *FfbZIP17* inhibited the ability of *F. fujikuroi* to resist oxidative stress, and other *FfbZIP* genes were not involved in the relevant regulation. *FfbZIP2*, *FfbZIP10*, *FfbZIP17*, *FfbZIP22*, *FfbZIP35*, and *FfbZIP44* inhibited the oxidative stress tolerance of *F. fujikuroi* under high oxidative stress, and other *FfbZIP* genes were not involved.

#### 2.6.3. Osmotic Stress Experiment

**Figure 6:**
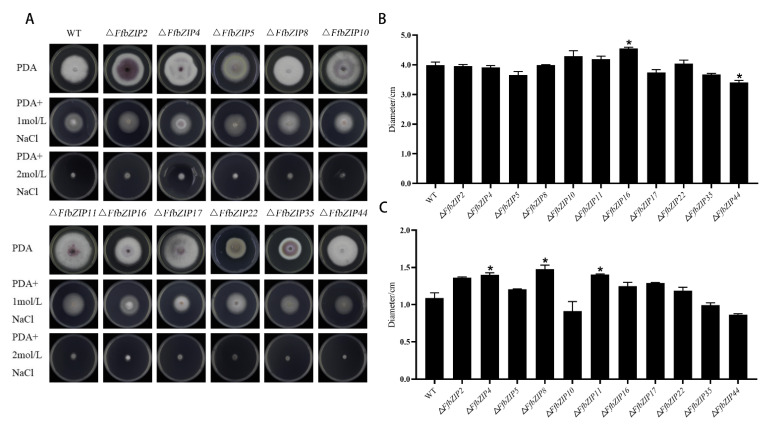
Osmotic stress experiment. (**A**) Growth of wild type and *FfbZIP* knockout mutants under NaCl osmotic stress. (**B**) Histogram of strain diameter under 1 mol/L NaCl osmotic pressure. Error bars represent means ± SEs. (**C**) Histogram of Histogram of strain diameter under 2 mol/L NaCl osmotic pressure. Error bars represent means ± SEs. * *p* < 0.05.

The osmotic stress experiment used NaCl to simulate a high osmotic pressure environment. Under 1 mol/L NaCl, only Δ*FfbZIP44* deletion mutant was smaller than the wild type, and it was significantly different from the wild type (*p* = 0.05) (Figure 6). Δ*FfbZIP16* was significantly larger than the wild type (*p* = 0.05), and the other deletion mutants had similar inhibitory effects to the wild type. Under 2 mol/L NaCl, Δ*FfbZIP4*, Δ*FfbZIP8* and Δ*FfbZIP11* were larger than the wild type, and there was a significant difference between the deletion mutants and the wild type at *p* = 0.05 level, but no difference between the wild type and all the deletion mutants at *p* = 0.01 level. The results indicated that *FfbZIP44* could enhance the resistance to osmotic stress of *F. fusarium*, under 1 mol/L NaCl, while other *FfbZIP* genes could not. Under 2 mol/L NaCl, *FfbZIP4*, *FfbZIP8* and *FfbZIP11* had a certain inhibitory effect on the osmotic tolerance of *F. fusarium*, while the other *FfbZIP* genes were without it.

#### 2.6.4. Cell Wall Selection Pressure Experiment

**Figure 7:**
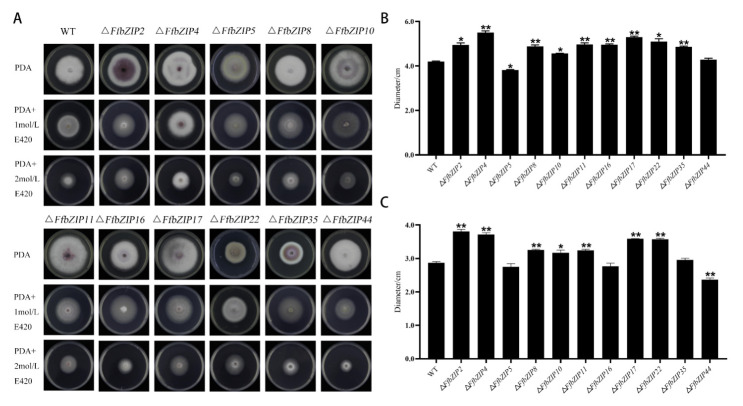
Cell wall selection pressure experiment. (**A**) Growth of wild type and *FfbZIP* knockout mutants under cell wall selection pressure. (**B**) Histogram of strain diameter under 1 mol/L E420 cell wall selection pressure. Error bars represent means ± SEs. (**C**) Histogram of strain diameter under 2 mol/L E420 cell wall selection pressure. Error bars represent means ± SEs, * *p* < 0.05, ** *p* < 0.01.

Cell wall selective stress experiment used sorbitol to simulate a cell wall stress environment. Under 1 mol/L sorbitol (E420), there was no significant difference between Δ*FfbZIP44* and the wild type, Δ*FfbZIP5* was significantly smaller than wild type (*p* = 0.05), and other mutants were significantly larger than wild type (Figure 7). Under 2 mol/L E420, the growth of Δ*FfbZIP5*, Δ*FfbZIP16*, and Δ*FfbZIP35* was similar to the wild type, and Δ*FfbZIP10* was significantly larger than the wild type (*p* = 0.05). Δ*FfbZIP44* was significantly smaller (*p* = 0.01) than the wild type, and the other deletion mutants were significantly larger than the wild type (*p* = 0.01). The results revealed that under 1 mol/L sorbitol (E420), *FfbZIP44* had no effect on the strain growth of *F. fujikuroi*, and the other *FfbZIP* genes were involved in the reverse regulation of the cell wall selective stress tolerance.

#### 2.6.5. Cellulose Utilization Experiment

**Figure 8:**
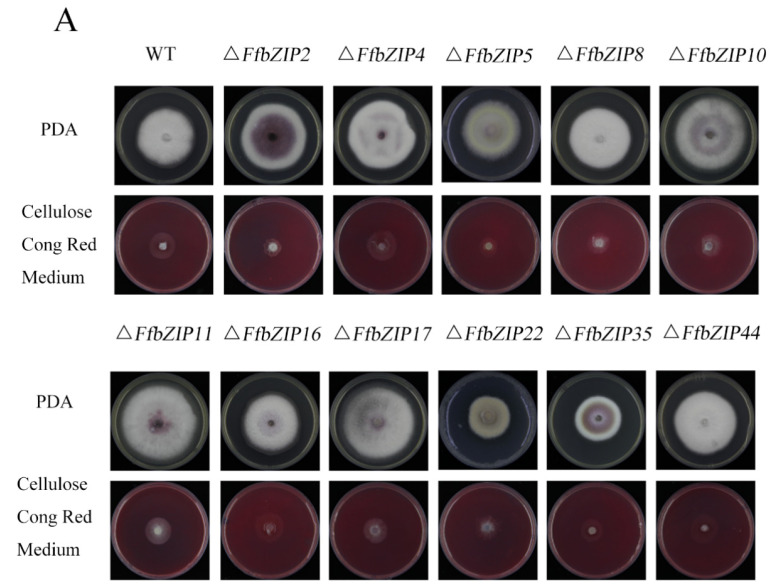

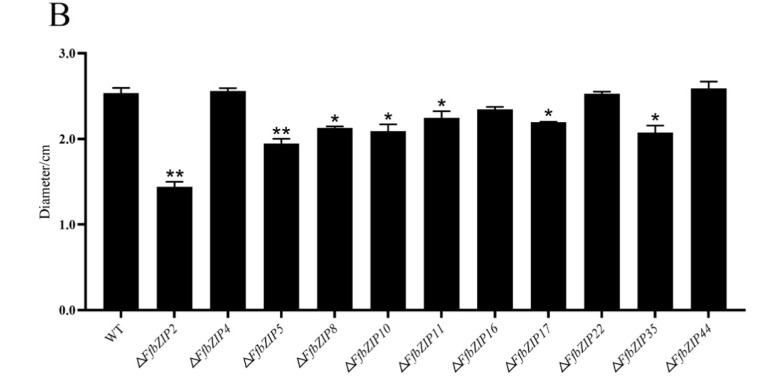
Cellulose utilization experiment. (**A**) Growth of wild type and *FfbZIP* knockout mutants in Cellulose Congo red medium. (**B**) Histogram of strain diameter under cellulose Congo red culture conditions. Error bars represent means ± SEs. * *p* < 0.05, ** *p* < 0.01.

Δ*FfbZIP2* and Δ*FfbZIP5* cultured on cellulose Congo red medium were much smaller than the wild type (*p* = 0.01), and Δ*FfbZIP8*, Δ*FfbZIP10*, Δ*FfbZIP11*, Δ*FfbZIP17* and Δ*FfbZIP35* were significantly smaller than the wild type (*p* = 0.05), while the rest were not (Figure 8). The results showed that *FfbZIP2*, *FfbZIP5*, *FfbZIP8*, *FfbZIP10*, *FfbZIP11*, *FfbZIP16*, *FfbZIP17* and *FfbZIP35* were involved in the absorption and utilization of cellulose, and the other *FfbZIP* genes had no effect.

#### 2.6.6. Cellophane Penetration Experiment

**Figure 9:**
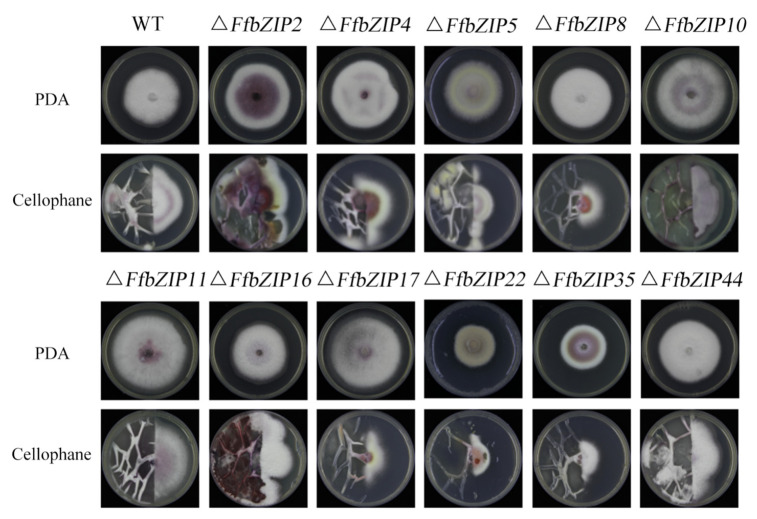
Determination of cellophane penetration ability of wild type and *FfbZIP* knockout mutants.

To clarify the ability of the deletion mutants to penetrate the plant cell wall, cellophane was used to simulate the plant cell wall. Δ*FfbZIP2*, Δ*FfbZIP4*, Δ*FfbZIP5*, Δ*FfbZIP16*, Δ*FfbZIP44* and wild type grew through cellophane, indicating their ability to penetrate plant cell walls, while Δ*FfbZIP8*, Δ*FfbZIP10*, Δ*FfbZIP11*, Δ*FfbZIP17*, Δ*FfbZIP22* and Δ*FfbZIP35* could not (Figure 9). The results revealed that *FfbZIP8*, *FfbZIP10*, *FfbZIP11*, *FfbZIP17*, *FfbZIP22*, and *FfbZIP35* played a decisive role in the penetration of *F. fujikuroi* infection into plant cell walls, while *FfbZIP2*, *FfbZIP4*, *FfbZIP5*, *FfbZIP16*, and *FfbZIP44* did not.

### 2.7. Pathogenicity Assay of FfbZIP Deletion Mutants

**Figure 10:**
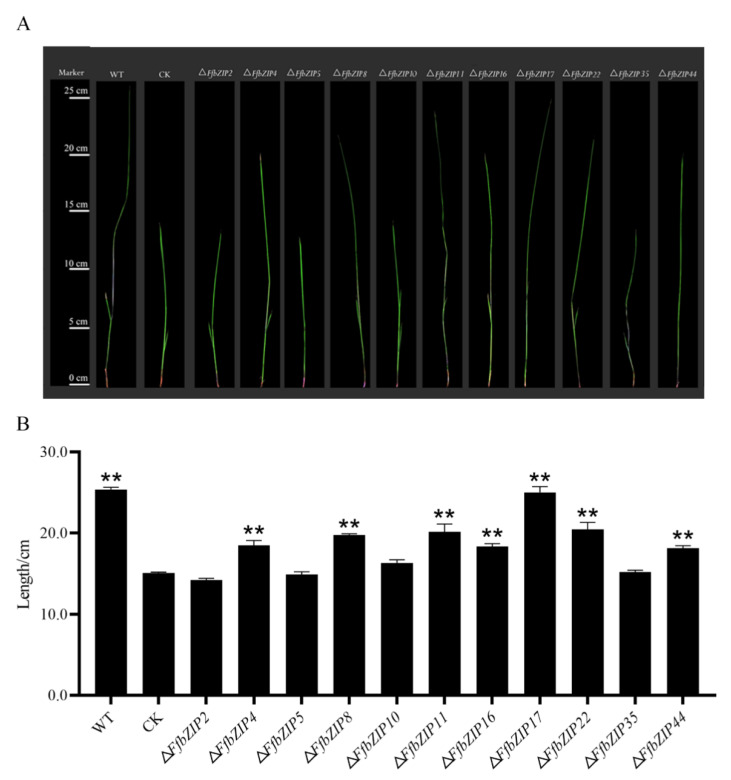
Pathogenicity observation of inoculated rice seedlings. (**A**) Pathogenicity alignment of *FfbZIP* knockout mutants and wild type. (**B**) Shoot length determination of rice seedlings inoculated with wild type and *FfbZIP* deletion mutants. CK stood for the shoot length of uninoculated rice seedlings, ** *p* < 0.01.

The pathogenicity assays used bakanae disease-susceptible rice varieties Zhongzao 39 and *FfbZIP* deletion mutants as test materials. Cultivated for 21 days after artificial inoculation, the growth of rice seedlings varies greatly (Figure 10A). The shoot length of rice seedlings was measured, and there was no significant difference in the growth of rice seedlings inoculated with Δ*FfbZIP2*, Δ*FfbZIP5,* and Δ*FfbZIP10* and uninoculated rice seedlings. Although the shoot length of rice seedlings inoculated with Δ*FfbZIP35* was not significantly different from that of uninoculated rice seedlings, the plants were thin, pale green and slightly whitish, showing typical symptoms of bakanae disease. The rice seedlings inoculated with Δ*FfbZIP4*, Δ*FfbZIP8*, Δ*FfbZIP11*, Δ*FfbZIP16*, Δ*FfbZIP22* and Δ*FfbZIP44* all became diseased, and the shoot length was significantly higher than that of the uninoculated rice seedlings, but significantly smaller than that of the wild-type seedlings. The difference was that the shoot length of rice seedlings inoculated with Δ*FfbZIP17* was not significantly different from that inoculated with wild type. The results showed that *FfbZIP2*, *FfbZIP5* and *FfbZIP10* were indispensable in the pathogenesis of *F. fujikuroi*. *FfbZIP4*, *FfbZIP8*, *FfbZIP11*, *FfbZIP16*, *FfbZIP22*, *FfbZIP35* and *FfbZIP44* had positive regulatory effects on the pathogenesis of *F. fujikuroi*, while *FfbZIP17* did not participate at all.

## 3. Discussion

In recent years, with the continuous improvement of genome databases, more and more TF families have been identified, such as bZIP [22], MYB [23], GATA [24], and PacC [25]. bZIP TFs are one of the most widely distributed and conserved proteins of eukaryotes. At present, a variety of fungal bZIP TF families have been identified. According to the published literature, a large number of bZIP TFs have been found in the genomes of many fungi such as *Alternaria* sp. [26], *Phytophthora sojae* [27], *Sclerotinia sclerotiorum* [28], *Aspergillus niger* [29], *F. graminearum* [30,31] *and F. pseudograminearum* [32]. Only a few bZIP TFs in fungi have been systematically identified, such as those in *M. oryzae* [9] and *F. graminearum* [33].

Currently, only one FfbZIP protein, MeaB, has been identified in *F. fujikuroi*. This protein cooperates with AreA to mediate nitrogen metabolite inhibition in *F. fujikuroi* [34]. This study focused on the bZIP TF family in *F. fujikuroi* and identified 44 FfbZIP proteins genome-wide for the first time. The number of bZIP TFs in *F. oxysporum* was 56. *U. virens*, *M. oryzae*, *F. graminearum*, *F. verticillium, F. solani*, *N. crassa* and *S. cerevisiae,* respectively, contained 28, 22, 21, 19, 17, 13 and 12 bZIP transcription factors, all less than *F. fujikuroi*. The results of phylogenetic analysis indicated that bZIP TFs in several common fungi were conserved in regulating the biological functions.

In the bZIP protein sequences of *F. fujikuroi*, in addition to the bZIP domain, there were some other domains (Table 2). This phenomenon was very common not only in plant species, but also in fungi. The function could be predicted through the domains FfbZIP TFs contained.

General features and species specificity are reflected in the exon-intron structures of *Arabidopsis*, Rice, Nematode, and Homo sapiens genes, whose average exon length all decreased with increasing intron number [55], while the exon length in the *FfbZIP* genes had no significant relationship with intron length and intron number. The features and species specificity among intron number, exon length and intron length of *Arabidopsis*, Rice, Nematode, and Homo sapiens genes were not available for *FfbZIP* genes.

Phylogenetic analysis of the total bZIP protein sequences of nine fungi including *F. fujikuroi* were divided into six clades according to the phylogenetic tree. Fungal bZIP TFs have diverse functions. By aligning bZIP TFs with model fungi, we predicted the function of FfbZIP proteins. Eight FfbZIP TFs were relatively specific in *F. fujikuroi*, indicating that these TFs may have different functions in *F. fujikuroi* relative to others.

Eleven *FfbZIP* deletion mutants were obtained by gene knockout. *FfbZIP4*, *FfbZIP8*, *FfbZIP10*, *FfbZIP16*, *FfbZIP17*, *FfbZIP35*, and *FfbZIP44* containing bZIP_YAP domain took part in stress response and osmotic stress response, and *FfbZIP35* containing PAP1 domain played a role in response to H_2_O_2_. The results of phenotype and pathogenicity analysis showed that the above *FfbZIP* genes were involved in the growth, stress response and invasion pathogenic process of *F. fujikuroi*, which was consistent with the previous prediction of domain functions (Table 3).

Though fungi have significant species diversity, due to their complex life cycle, unknown growth environment and other factors, researchers continue to pose new questions. Due to the large number of bZIP TFs, the signaling mechanism of each subfamily and the cross-function between subfamilies need further study. It is expected that researchers will isolate more bZIP TFs, so as to further explore their roles in hormone signaling, anti-stress pathways, pathogenicity, as well as the regulatory mechanism of energy metabolism pathways, and further improve the functional research of bZIP TFs.

In view of the diversity and importance of the biological functions of bZIP TFs, gene cloning technology can be combined with transcriptomics, proteomics, and other technical means to expand the research field of bZIP TFs, strengthen the study of bZIP in a wider range of species, and explore more theoretical and applied values. In addition, more attention needs to be paid to the response regulation pathway of bZIP TFs and explore the dynamic process of the bZIP network regulation mechanism using fungal tracer technology. By applying system biology methods and exploring the regulatory network of the upstream and downstream genes of bZIP TFs, the network regulatory mechanism of bZIP in response to environmental stress will be revealed. In general, a comprehensive understanding of the distribution, classification, structure, and function of bZIP TFs, and the realization of their global and dynamic research will be the future research direction.

## 4. Materials and Methods

### 4.1. Identification of bZIP Transcription Factors in F. fujikuroi

The protein data of *F. fujikuroi* (IMI 58289) was downloaded from the National Center for Biological Information (http://www.ncbi.com, accessed on 8 June 2022), and the gene sequences with PF00170 (bZIP_1), PF07716 (bZIP_2) and PF03131 (bZIP_Maf) were searched and aligned by the HMMER software using the Hidden Markov Model (HMM) profile of Pfam [56,57]. The E value threshold was set to be ≤500, and the HMM search operation was performed. The amino acid sequences of the bZIP transcription factor of *F. graminearum*, *F. oxysporum*, *Saccharomyces cerevisiae*, *Ustilaginoidea virens*, *Neurospora crassa*, *Magnaporthe oryzae*, *F.* *verticillioides* and *F.* *solani* were downloaded from the Fungal Transcription Factor Database (http://ftfd.snu.ac.kr/, accessed on 1 June 2022) as the reference sequences. 21, 56, 12, 27, 13, 22, 19 and 17 bZIP protein sequences were obtained, respectively [58,59]. These sequences were used as queries for TBLASTN searches (E-value cutoff less than 1 × 10^−5^) against the *F. fujikuroi* genome. We removed repeated sequences and kept similar sequences. The presence of typical bZIP domains in predicted protein sequences were verified through CD-Search (http://www.ncbi.nlm.nih.gov/Structure/cdd/wrpsb.cgi/, accessed on 5 June 2022) and Multiple Em for Motif Elicitation (MEME) (https://meme-suite.org/meme/tools/meme/, accessed on 5 June 2022), with an E-value threshold of 0.01 [9]. After deleting protein sequences that did not contain bZIP domains, the remaining sequences were considered as bZIP protein candidates in *F. fujikuroi* for further analysis.

### 4.2. Structural Analysis of bZIP Transcription Factors

The intron and exon structure information of *FfbZIP* genes was annotated in the genome.gff file (GCF_900079805.1_Fusarium_fujikuroi_IMI58289_V2_genomic.gff) downloading from the National Center for Biological Information (http://www.ncbi.com/, accessed on 9 June 2022). The Gene Structure Display Server (GSDS) 2.0 (http://gsds.gao-lab.org/, accessed on 2 June 2022) was used to annotate the intron and exon structure information [60]. The protein sequences of annotated DNA-binding domains and other functional domains from CD-Search screening were graphically displayed on full-length genes using TBtools [61]. Multiple sequence alignments of the amino acid sequences of bZIP were aligned using the DNAMAN [62].

### 4.3. Phylogenetic Analysis of bZIP Transcription Factors

Phylogenetic analysis of *F. fujikuroi* with *F. graminearum*, *F. oxysporum*, *Saccharomyces cerevisiae*, *Ustilaginoidea virens*, *Neurospora crassa*, *Magnaporthe oryzae*, *F. verticillioides* and *F. solani* was performed using iqTREE [63], and the optimal model calculated by Model Finder was used to complete the verification of 1000 bootstraps [64]. Phylogenetic analysis results were visualized by iTOL software [65].

### 4.4. Generation of FfbZIP Gene Deletion Mutants

The principle of homologous replacement was used to knock out the *FfbZIP* genes, and the *FfbZIP* genes were replaced with the hygromycin resistance gene. PCR technology was used for DNA fragment amplification and ligation. The principle was shown in Supplementary Figure S4. The upstream/downstream fragment of the target gene with the hygromycin resistance gene linkers were amplified by F1 + R/R1 + F primers, and the hygF/hygR hygmycin resistance genes were amplified by universal hygmycin resistance gene primers. The three fragments were directly connected by PCR technology, and then the PCR products were directly amplified by F2 + H3/R2 + H2 primers to obtain upstream and downstream fragments of the target gene with partially overlapping hygromycin resistance genes respectively.

Using *F. fujikuroi* IMI 58289 genomic DNA as a template, 44 primers (Supplementary Table S1) were used to amplify the upstream and downstream sequences of 11 genes, including *FfbZIP2*, *FfbZIP4*, *FfbZIP5*, *FfbZIP6*, *FfbZIP10*, *FfbZIP11*, *FfbZIP16*, *FfbZIP17*, *FfbZIP22*, *FfbZIP35* and *FfbZIP44*, about 2 kb, for gene knockout. Using the LiGFP plasmid as a template, the hygromycin resistance gene HygR was amplified. 50 μL PCR reaction system: 25 μL Mix enzyme, 2 μL *F. fujikuroi* DNA template, 3 μL upstream/downstream primers (F1 + R/F + R1), 17 μL H_2_O. PCR reaction program: pre-denaturation at 94 °C for 2 min; 30 cycles of 94 °C for 30 s, 58 °C for 30 s, and 72 °C for 2 min; 72 °C for 10 min; 16 °C for 10 min. The PCR products were electrophoresed on agarose gels and recovered by cutting the gel using the TIANgel Midi Purification Kit (Tiangen Biotech (Beijing) Co., Ltd., Beijing, China).

The upstream and downstream of the target gene and the hygromycin resistance gene three fragments were connected by PCR reaction. 25 μL PCR reaction system: 12.5 μL Mix enzyme, 1 μL upstream and downstream fragments of target gene, 3 μL hygromycin resistance gene fragment, 7.5 μL H_2_O. PCR reaction program: pre-denaturation at 94 °C for 2 min; 94 °C for 30 s, 60 °C for 10 min, 72 °C for 5 min, 9 cycles of these conditions; 72 °C for 10 min; 16 °C for 10 min. We used the above PCR products as the DNA template to directly repeat the PCR operation in the previous steps, changed the primers to F2 + H3/H2 + R2, and kept other conditions unchanged. The PCR products were electrophoresed on agarose gels and recovered by cutting the gel using the TIANgel Midi Purification Kit.

Fungal protoplasts of the wild-type strain FF43 were directly transformed by the standard PEG-mediated method [66]. The fungus grown on the PDA (containing 200 μg/mL hygromycin) plate was transferred to a new PDA (containing 200 μg/mL hygromycin) plate, cultured at 28 °C for 5 days, using the KAPA3G Plant PCR Kit (Merck & Co. Inc., Kenilworth, NJ, USA) PCR detection. In addition, we set primers on the upstream and downstream of the target gene to amplify the target fragment by f/r PCR. If the wild-type and transformant-amplified fragments had different lengths and no identical bands, it meant that the hygromycin resistance gene had replaced the target gene, that is, the target gene had been knocked out.

### 4.5. Phenotype Assays

(a) Strain growth rate observation. The fungi block with 7 mm diameter was cut from the outer periphery of the wild-type strain and deletion mutants cultured for 5 days. We cultured these fungi blocks in PDA (Biological Technology Co., Shanghai, China Zhaorui) medium with natural light at 28 °C, measured the diameter of the strains every 24 h for 7 days and observed the morphological characteristics of the strains (color, shape, growth of aerial hyphae, etc.). Analysis of variance (ANOVA) was used to determine the growth between wild-type and mutant strains (Statistical Product Service Solutions (SPSS)). Each strain was inoculated in three replicates [67].
(b) Stress sensitivity test. Oxidative stress experiment: the volume percentages concentration of 0.1%, 0.25%, and 0.5% of H_2_O_2_ (Aladdin Biochemical Technology Co., Ltd., Shanghai, China) were added to the PDA medium to prepare H_2_O_2_ PDA mediums with different concentrations; osmotic stress experiment: different concentrations of NaCl (National Pharmaceutical Group Chemical Reagent Co., Ltd., Beijing, China) were added to the PDA medium to prepare PDA mediums containing NaCl concentrations of 1 mol·L^−1^ and 2 mol·L^−1^; cell wall selection pressure experiment: sorbitol PDA mediums with 1 mol·L^−1^ and 2 mol·L^−1^ concentrations were prepared by adding sorbitol; cellulose utilization experiment: cellulose Congo red mediums (Zhaorui Biological Technology Co., Shanghai, China) were used. The 7 mm diameter fungi blocks of *F. fujikuroi* cultured for 5 days were transferred in the center of the each medium. They were cultured in a 28 °C incubator for 5 days, we observed and measured the strain size and took pictures to record. Analysis of variance was used to determine the growth between wild-type and mutant strains (Data Processing System (DPS)). The above experiments were repeated three times for each treatment [68,69].
(c) Cellophane penetration experiment: tested the penetration ability of *F. fujikuroi* with cellophane (Dingguo Changsheng Biotechnology Co., Ltd., Beijing, China) equivalent to plant cell wall. Took 5 mm fungi blocks of mutants and wild type strains of *F. fujikuroi* and placed the mycelium side down in the center of a 9 mm PDA medium. We sterilized a semicircular cellophane with a radius of 4 cm, and stock it on the medium with 3/4 fungi block covered. Each strain was inoculated in three replicates. Analysis of variance was used to determine the growth between wild-type and mutant strains (DPS). Strains were cultured in an incubator at 28 °C for 5 days and photographed for recording [70].

### 4.6. Strains and Inoculation Experiments

*F. fujikuroi* strain FF43 was isolated from infected rice spikelet in Zhejiang, China. The tested rice variety was Zhongzao 39, which was a bakanae disease-susceptible variety. The artificial inoculation of the bakanae disease pathogen referred to Zhao Yuan [71]. Rice seeds were sterilized with 3% H_2_O_2_ for 2 h, soaked for 2 days, and germinated for 1 day. The bakanae disease pathogen and sprouted seeds were inoculated into sterilized vermiculite respectively, and the vermiculite without pathogen was used to grow rice as a control. The treated rice seeds were placed in an incubator with 12 h of light and 12 h of darkness at 30 °C. They were watered each morning and evening, two milliliters at a time. There were three replicates for each treatment. DPS was used to analyze the growth differences of rice seedlings inoculated by wild-type or deletion mutant strains.

## 5. Conclusions

We identified 44 bZIP TFs by analyzing the whole genome data of *F. fujikuroi*. Through gene structure analysis, phylogenetic analysis of FfbZIP TFs, and the phenotype and pathogenic analysis of deletion mutants, it was confirmed that these TFs took part in *F. fujikuroi* growth, nutrient absorption and utilization, stress resistance and pathogenicity. Our research results provided help for an in-depth understanding of the regulatory mechanism of *F. fujikuroi* growth, stress management and pathogenicity, and provided a reference for the research of other phytopathogenic fungi.

## Figures and Tables

**Table 1:** List of bZIP TF family identified in the *F. fujikuroi* genome.

Gene Name	Locus	Gene Symbol	Gene ID	Chromosome	Location	Protein (aa)	ORF (bp)
*FfbZIP1*	XP_023423521.1	FFUJ_02084	35395567	chrom01	19507..20852	389	1170
*FfbZIP2*	XP_023423830.1	FFUJ_01766	35395250	chrom01	994959..996723	570	1713
*FfbZIP3*	XP_023423951.1	FFUJ_01635	35395120	chrom01	1400129..1402170	592	1779
*FfbZIP4*	XP_023425446.1	FFUJ_01507	35394992	chrom01	1910261..1911646	362	1089
*FfbZIP5*	XP_023424500.1	FFUJ_01033	35394518	chrom01	3500633..3502539	612	1839
*FfbZIP6*	XP_023425433.1	FFUJ_00012	35393497	chrom01	6509845..6511070	389	1170
*FfbZIP7*	XP_023426226.1	FFUJ_04663	35398144	chrom02	2022715..2024097	313	942
*FfbZIP8*	XP_023426254.1	FFUJ_04631	35398112	chrom02	2122298..2124080	571	1716
*FfbZIP9*	XP_023427120.1	FFUJ_04440	35397921	chrom02	2838516..2839721	383	1152
*FfbZIP10*	XP_023426709.1	FFUJ_04132	35397613	chrom02	3893261..3894237	277	834
*FfbZIP11*	XP_023426718.1	FFUJ_04122	35397603	chrom02	3926173..3927310	333	1002
*FfbZIP12*	XP_023427258.1	FFUJ_02097	35395580	chrom03	34849..35802	317	954
*FfbZIP13*	XP_023427633.1	FFUJ_02504	35395986	chrom03	1197966..1198797	259	780
*FfbZIP14*	XP_023427873.1	FFUJ_02765	35396247	chrom03	2066960..2068584	460	1383
*FfbZIP15*	XP_023427987.1	FFUJ_02890	35396372	chrom03	2501702..2502858	356	1071
*FfbZIP16*	XP_023428133.1	FFUJ_03047	35396529	chrom03	3055572..3057068	456	1371
*FfbZIP17*	XP_023428386.1	FFUJ_03322	35396804	chrom03	3964224..3965739	406	1221
*FfbZIP18*	XP_023428399.1	FFUJ_03337	35396819	chrom03	4023514..4025190	558	1677
*FfbZIP19*	XP_023428821.1	FFUJ_03431	35396913	chrom03	4359051..4359823	237	714
*FfbZIP20*	XP_023428723.1	FFUJ_03684	35397165	chrom03	4988201..4989780	485	1485
*FfbZIP21*	XP_023429592.1	FFUJ_13014	35406470	chrom04	156410..157306	268	807
*FfbZIP22*	XP_023429297.1	FFUJ_13410	35406864	chrom04	1320310..1321873	281	846
*FfbZIP23*	XP_023429736.1	FFUJ_14626	35408007	chrom04	2666927..2667729	249	750
*FfbZIP24*	XP_023429799.1	FFUJ_14713	35408093	chrom04	2879385..2880540	315	948
*FfbZIP25*	XP_023430031.1	FFUJ_06701	35400178	chrom05	3676..4515	279	840
*FfbZIP26*	XP_023430081.1	FFUJ_06752	35400229	chrom05	130034..131519	478	1437
*FfbZIP27*	XP_023430603.1	FFUJ_07310	35400787	chrom05	1704249..1706126	526	1581
*FfbZIP28*	XP_023430915.1	FFUJ_07653	35401130	chrom05	2919638..2920792	384	1155
*FfbZIP29*	XP_023431104.1	FFUJ_07958	35401435	chrom05	3847775..3849637	547	1644
*FfbZIP30*	XP_023431611.1	FFUJ_05423	35398902	chrom06	236543..237482	271	816
*FfbZIP31*	XP_023431622.1	FFUJ_05435	35398914	chrom06	270287..271705	395	1188
*FfbZIP32*	XP_023431638.1	FFUJ_05454	35398933	chrom06	309593..310621	325	978
*FfbZIP33*	XP_023433931.1	FFUJ_05599	35399078	chrom06	747268..748842	255	768
*FfbZIP34*	XP_023432344.1	FFUJ_06219	35399696	chrom06	2879412..2880716	303	912
*FfbZIP35*	XP_023432438.1	FFUJ_06324	35399801	chrom06	3250263..3252143	589	1770
*FfbZIP36*	XP_023432582.1	FFUJ_06482	35399959	chrom06	3687836..3689435	478	1437
*FfbZIP37*	XP_023433931.1	FFUJ_08968	35402442	chrom07	478942..479843	255	768
*FfbZIP38*	XP_023434584.1	FFUJ_12383	35405839	chrom08	1819337..1819834	165	498
*FfbZIP39*	XP_023434615.1	FFUJ_12415	35405871	chrom08	1903509..1904411	300	903
*FfbZIP40*	XP_023434864.1	FFUJ_12679	35406135	chrom08	2609336..2611017	507	1524
*FfbZIP41*	XP_023435893.1	FFUJ_09363	35402832	chrom09	2512271..2513912	497	1494
*FfbZIP42*	XP_023436813.1	FFUJ_10801	35404265	chrom10	1589255..1591402	465	1398
*FfbZIP43*	XP_023437623.1	FFUJ_11604	35405065	chrom11	1056054..1056872	256	771
*FfbZIP44*	XP_023437959.1	FFUJ_11961	35405421	chrom11	1910792..1912552	530	1593

**Table 2:** FfbZIP transcription factor domains and their function.

Domain	Gene	Function	Reference
bZIP domain	*FfbZIP5*, *FfbZIP7*, *FfbZIP12*, *FfbZIP28*, *FfbZIP42*, *FfbZIP43*	Regulates a diverse range of cellular processes, including cell survival, learning and memory, lipid metabolism, and cancer progression; also plays an important role in the response to stimuli or stress signals such as cytokines, genotoxins, or physiological stress	[35,36]
bZIP_YAP domain	*FfbZIP1*, *FfbZIP3*, *FfbZIP4*, *FfbZIP6*, *FfbZIP8*, *FfbZIP9*, *FfbZIP10*, *FfbZIP13*, *FfbZIP14*, *FfbZIP15*, *FfbZIP16*, *FfbZIP17*, *FfbZIP19*, *FfbZIP20*, *FfbZIP21*, *FfbZIP23*, *FfbZIP24*, *FfbZIP26*, *FfbZIP29*, *FfbZIP30*, *FfbZIP31*, *FfbZIP32*, *FfbZIP34*, *FfbZIP35*, *FfbZIP36*, *FfbZIP40*, *FfbZIP41*, *FfbZIP44*	May be involved in stress response, cadmium stress response, osmotic stress response, iron metabolism and arsenic detoxification	[37]
Bzip_u1 domain	*FfbZIP2*	uncharacterized	[38]
bZIP_GCN4 domain	*FfbZIP11*, *FfbZIP38*	In amino acid-deficient cells, GCN4 is upregulated, leading to transcriptional activation of genes encoding amino acid biosynthesis enzymes	[39]
bZIP_Zip1 domain	*FfbZIP18*, *FfbZIP22*	Zip1 is required for the production of key proteins involved in sulfur metabolism and also plays a role in the cadmium response	[40]
bZIP_ATF2 domain	*FfbZIP25*, *FfbZIP27*, *FfbZIP37*, *FfbZIP39*	In response to stress, ATF-2 activates multiple genes, including cyclin A, cyclin D, and c-Jun. ATF-2 also plays a role in the DNA damage response independent of its transcriptional activity	[41]
bZIP_HAC1-like domain	*FfbZIP33*	Plays a key role in the unfolded protein response (UPR)	[42]
VirB10 domain	*FfbZIP4*, *FfbZIP29*	Different domains of VirB10 in Agrobacterium coordinately regulate T fimbriae formation or secretion channels	[43]
Smc domain	*FfbZIP5*	This domain is present in chromosome segregation ATPases that regulate the cell cycle, cell division and chromosome segmentation; in vertebrates it functions to regulate genome structure during interphase and cell division	[44,45,46]
PTZ00449 domain	*FfbZIP10*	uncharacterized	[47]
PTZ00108 domain	*FfbZIP37*	uncharacterized	[48]
DUF3425 domain	*FfbZIP3*, *FfbZIP9*, *FfbZIP15*, *FfbZIP16*, *FfbZIP23*, *FfbZIP29*, *FfbZIP32*, *FfbZIP36*, *FfbZIP40*, *FfbZIP41*	uncharacterized	[49]
KLF1_2_4_N domain	*FfbZIP17*	Members of the KLF family can act as activators or repressors of transcription depending on the context of the cell and promoter, regulating various cellular functions such as proliferation, differentiation and apoptosis, as well as development and homeostasis of several types of tissues	[50]
PAP1 domain	*FfbZIP35*	Regulation of antioxidant gene transcription in response to H_2_O_2_	[51]
Atf1_OSA	*FfbZIP27*	This domain is found in the transcription factor Aft1 which is required for a wide range of stress responses. The OSA domain has been shown to be involved in the osmotic stress response.	[52]
Atf1_HRA	*FfbZIP27*	This domain is found in the transcription factor Aft1 which is required for a wide range of stress responses. The HRA domain is involved in meiotic recombination. It has been shown to be necessary and sufficient to activate recombination.	[52]
Atf1_HRR	*FfbZIP27*	This domain is found in the transcription factor Aft1 which is required for a wide range of stress responses. The HRR domain is involved in meiotic recombination. It has been shown to be necessary and sufficient to repress recombination.	[52]
Ank_2	*FfbZIP6*	Ankyrin repeats (3 copies).	[53]
PHA03247	*FfbZIP18*, *FfbZIP21*	large tegument protein UL36; Provisional.	[54]

**Table 3:** The biological function and pathogenicity of *FfbZIP* genes.

	Gene	Vegetative Growth	Oxidative Stress	Osmotic Stress	Cellulose Utilization	Cell Wall Selective Pressure	Cellophane Penetration		Patablethogenicity
Function									
*FfbZIP2*		\ *	− *	−	+ *	−		\	+
*FfbZIP4*		\	+	−	\	−		\	+
*FfbZIP5*		\	\	+	+	+		\	+
*FfbZIP8*		−	\	−	+	−		+	+
*FfbZIP10*		−	−	−	+	−		+	+
*FfbZIP11*		−	\	−	+	−		+	+
*FfbZIP16*		\	\	−	\	−		\	+
*FfbZIP17*		−	−	−	+	−		+	\
*FfbZIP22*		+	−	−	\	−		+	+
*FfbZIP35*		\	−	−	+	−		+	+
*FfbZIP44*		−	−	+	\	+		\	+

* “\” meant no effect, “+” meant forward regulation, “−” meant reverse regulation.

## Data Availability

The data presented in this study are available in insert article or supplementary material here.

## Supplementary Materials

The following supporting information can be downloaded at: https://www.mdpi.com/article/10.3390/ijms23126658/s1.
