# Supplementary material for: Genome-Wide Identification and Functional Analysis of the bZIP Transcription Factor Family in Rice Bakanae Disease Pathogen, Fusarium fujikuroi
Source: Int J Mol Sci. 2022 Jun 15;23(12):6658. doi: 10.3390/ijms23126658 (PMC9223689; doi:10.3390/ijms23126658)
Supplement: Supplementary file 1 [file ijms-23-06658-s001.zip › ijms-1735552-SI.pdf]

Supplementary Materials:

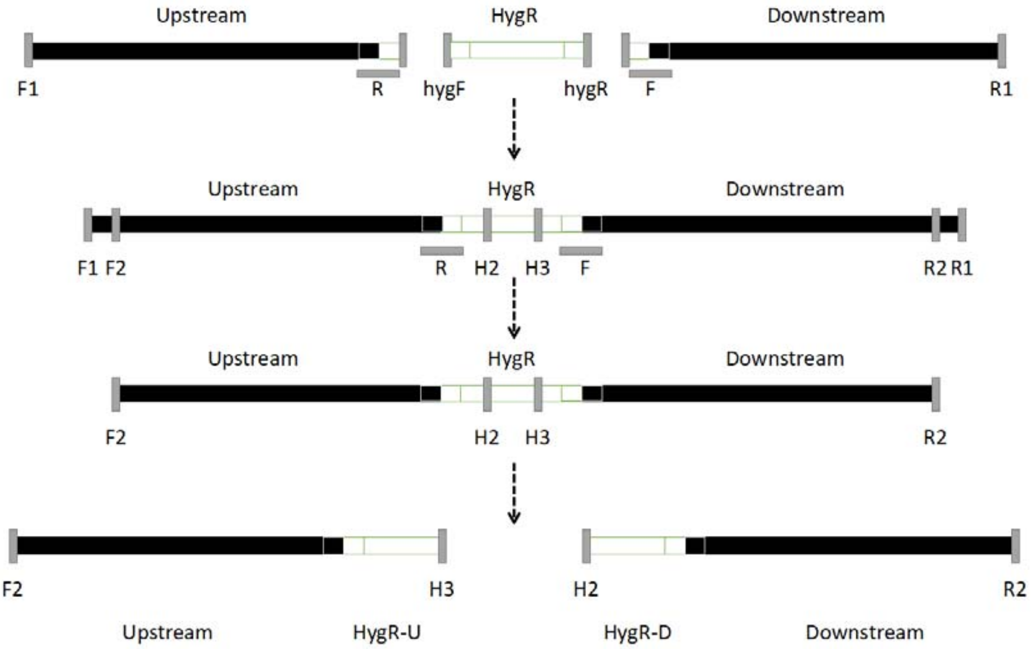

Figure S1. Schematic diagram of gene knockout process.

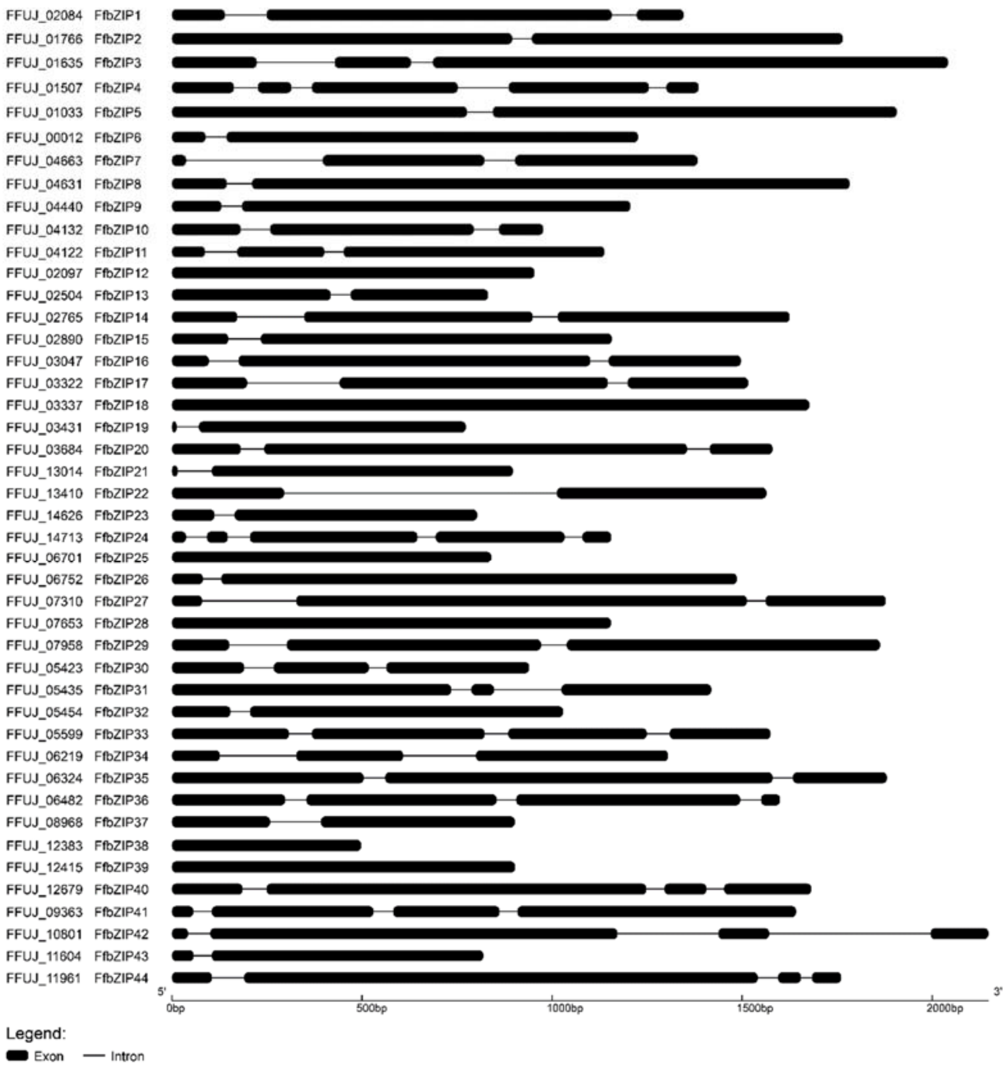

Figure S2. The relationship between *FfbZIP* exon length, intron length and intron number. A Relationship between exon length and introns number. B Relationship between intron length and introns number. C Relationship between exon and intron length.

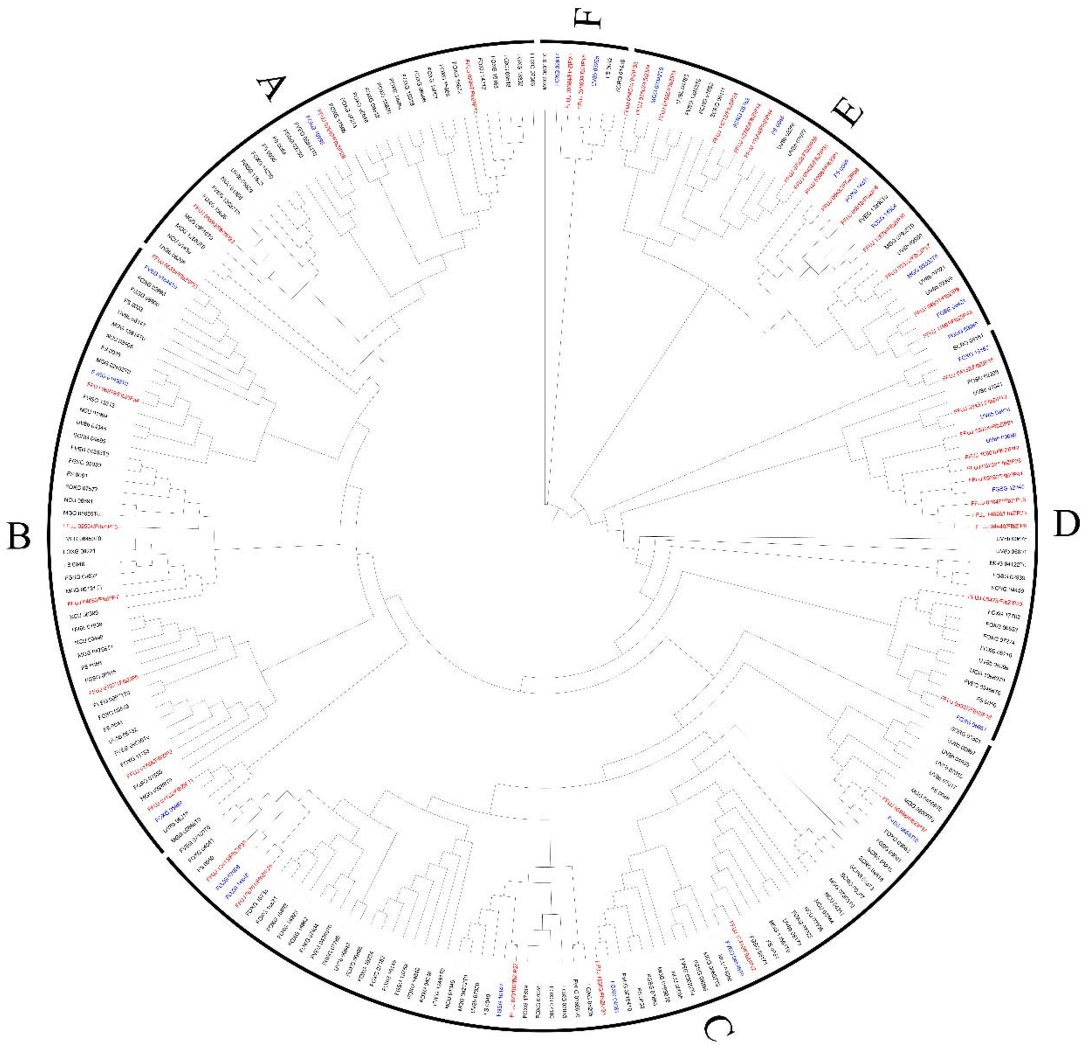

Figure S3. Phylogenetic analysis of bZIP transcription factors in nine fungal species.

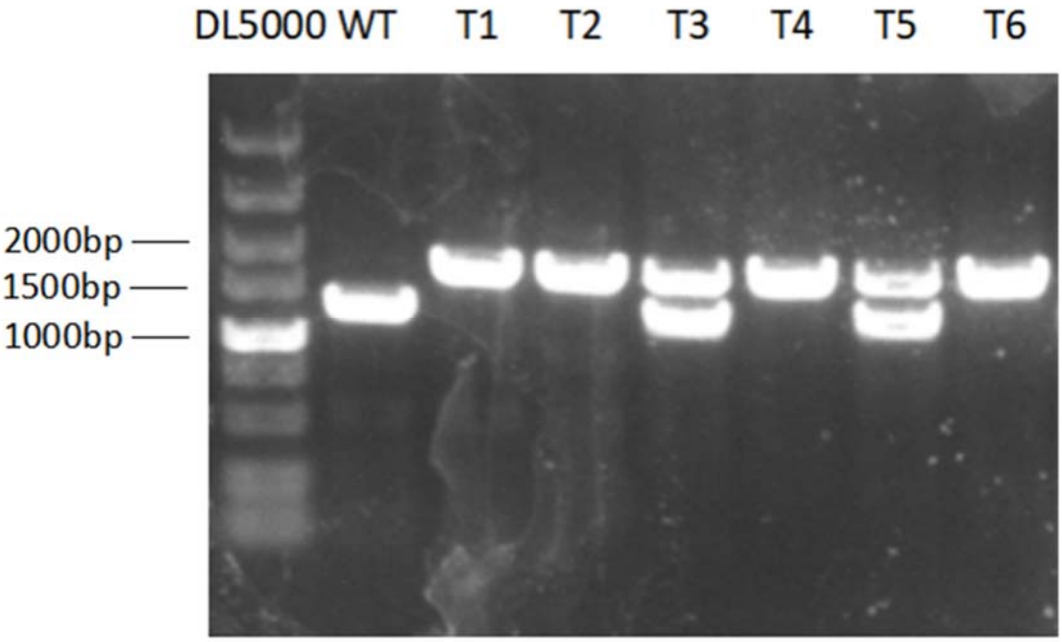

Figure S4. Screening and identification of *FfbZIP* transcripts. Using f/r primers detected the presence or absence of HygR or the target gene at a specific position. The amplified band of the target gene was 1200 bp, and the HygR amplified band was 1500 bp. Only one 1200bp band could be amplified in the wild type. The amplification of two bands indicated that the HygR was inserted randomly and did not replace the target gene. The transformants that only amplified the 1500bp band were positive.

Table S1. List of primers used for the construction of *FfbZIP* gene deletion mutants.

Primer name	Sequence
H2	GGTAAATAGCTGCGCCGATGGTT
H3	GAACATCGCCTCGCTCCAGTCA
hypF	TCGACAGAAGATGATATTGAAGGAG
hypR	GTTAAGTGGATCCGGCATCT
DbZIP2R1	GCGTAGACATGGTCTCGAGG
DbZIPR2	GCGCGGTTATGGTTGACATC
UbZIP2F1	ACGATTATAGCAGCGTGGCA
UbZIP2F2	TCGGAAAGCATACCAGCCAG
UbZIP2R	TCAATATCATCTTCTGTCGAGTTGTTGTATCAGTTCGGCG
DbZIP2F	AGATGCCGGATCCACTTAACGTGTGAATAGGAGTTTGCTT
DbZIP4R1	TCTGTTCGGACGACAAGGTG
DbZIP4R2	AGGGGTAAACGGATCGAGGA
UbZIP4F1	GCCGTATTTTTGCCAGCGAT
UbZIP4F2	CCACCCTTTCCCCAAGACTC

Primer name	Sequence
UbZIP4R	TCAATATCATCTTCTGTCGAACTGGTCGCCTATGAGAGGT
DbZIP4F	AGATGCCGGATCCACTTAACACGAAGACACGAATCAAGAA
DbZIP5R1	CCATTGGTGTAGCAGCCGTA
DbZIP5R2	CACGGCTCATCGCTATAGCA
UbZIP5F1	AGACATCTCCAACTGTGCCG
UbZIP5F2	AACTCGCGAGACAAGACCTG
UbZIP5R	TCAATATCATCTTCTGTCGATGTGGTGATGTGAGATTGAG
DbZIP5F	AGATGCCGGATCCACTTAACAGCCAGTAACTAACAACGTA
DbZIP8R1	TGCTGAAGATGCTCATTTGG
DbZIP8R2	GCAGTCTGCCAAAGTTCCTC
UbZIP8F1	CACGATCCCCTTCTCCCATG
UbZIP8F2	ACATGGCGATGGTCGAGTTT
UbZIP8R	TCAATATCATCTTCTGTCGAGGTGGCTGGATTTTAAATGA
DbZIP10F	AGATGCCGGATCCACTTAACACCAATAGGGCAGCTGGCAT
DbZIP10R1	CTCCCGGCAACAGTATCATT
DbZIP10R2	TCCAAATCCTCCCAGAACAG
UbZIP10F1	TCTGCTCTGCTAATAGCGCC
UbZIP10F2	CGTTGACTCTCCACCGTTGA
UbZIP10R	TCAATATCATCTTCTGTCGAACATGGACATTGTGAAGTTG
DbZIP11F	AGATGCCGGATCCACTTAACCCGGCATGCAATAATGGATT
DbZIP11R1	TGCTAGATCCCGGTTATTCG
DbZIP11R2	CGGTGTCCCCATCGATATAC
UbZIP11F1	GCAGACAGGGCAGGAAAGAT
UbZIP11F2	CGAGTGGGCTGAATCCAGTT
UbZIP11R	TCAATATCATCTTCTGTCGAAAGCCATGGTGACTGGGGAT
DbZIP16F	AGATGCCGGATCCACTTAACGATAATACACAACTACGGAC
DbZIP16R1	TCCAGCGAACCGGTACTAAC
DbZIP16R2	AAACAGCTGGGACAACAAGG
UbZIP16F1	GTGACCACAACCACATTGGC
UbZIP16F2	TCTCGGCTGAAGGTTGTGAC
UbZIP16R	TCAATATCATCTTCTGTCGACACCCATAATATTCCATTGG
DbZIP17F	AGATGCCGGATCCACTTAACACTTGGAGCTCGAACAAACC
DbZIP17R1	TGCTGGATATGCGATACTGG

DbZIP17R2	GGAATCATGCTTTGGGTGTC
UbZIP17F1	ATGTGGTTGTGTAGAGGCCG
UbZIP17F2	TCAAATTCCCTCGACACGGG
UbZIP17R	TCAATATCATCTTCTGTCGACTTGTCTGCCATTACCCAGA
DbZIP22F	AGATGCCGGATCCACTTAACATCCGTCGTATAGCGTCGGG
DbZIP22R1	CACGCTGAGCATAAAGACGA
DbZIP22R2	ATCAGAAGTTCGGGTGATGG
UbZIP22F1	TATTCGGCTCAGCGCAAGAA
UbZIP22F2	TGGCTTGGCTGGTCGTATTT
UbZIP22R	TCAATATCATCTTCTGTCGAGAAGTTTGACATATTGGTGG
DbZIP35F	AGATGCCGGATCCACTTAACGGCCAGGCTTATTATGAACA
DbZIP35R1	TCGCTGTTGGTCTGAGTCAC
DbZIP35R2	GCCGTCTTGAGATAGCCCTC

Primer name	Sequence
UbZIP35F1	GTAAGCGACATACTCCGGGG
UbZIP35F2	GCAGCCGTTGAGATGCAAAA
UbZIP35R	TCAATATCATCTTCTGTCGACGCGAAATATGAAACGGGCG
DbZIP44F	AGATGCCGGATCCACTTAACGATAGGAAGAAGCGGGGACG
DbZIP44R1	CGGATCCATGGTGAGCTCTC
DbZIP44R2	AGTGGGGGAGAATCAGACGA
UbZIP44F1	TGTGTACGCTGCTCTCAAGG
UbZIP44F2	AGTCCACGGACCTCTAAGCT
UbZIP44R	TCAATATCATCTTCTGTCGATTCCGAGGTAGAAGAACTCA
HPH-F	CTATTGCATCTCCCGCC
HPH-R	CCATACAAGCCAACCACG
bZIP2f	TCCCGATTTTCTGCCTGGTT
bZIP2r	ACGCCTAATCCCATCCCAATC
bZIP4f	CAGGAGCAAAACCACGACAA
bZIP4r	CGCGCCCTGTACAGTAATATG
bZIP5f	CGTACCCGTTTCGTCCTTCA
bZIP5r	GCGTGAGCAACATGTCTGGA
bZIP8f	ATACGGAGCATAATCGGCGG
bZIP8r	GGCAAGCACAGGAATTGTGAC
bZIP10f	AGCCTCTCAGTTCGTTTGGG

bZIP10r	CAAAACCCGCCATGCTATGC
bZIP11f	CCCAGGACCTTAACGCATCA
bZIP11r	TTCATCAATGGGTACAGCGAATG
bZIP16f	AACTCGACCTCCTTGACACC
bZIP16r	GTCATCAACCTGTCTTGTAGCAC
bZIP17f	CTTGACGTTCCACCTCGTCA
bZIP17r	ATAGCAGCTCTTCAGGCGTC
bZIP22f	CGGAACTGGTACCGAACGAA
bZIP22r	CCTCACGAAACCTCGCGATA
bZIP35f	GTCTGGCTCTTTGTCCCCTC
bZIP35r	TGACGGAGGTAAATCAAGCG
bZIP44f	CATCTCGATCTCTGGGCGAA
bZIP44r	GGCTTTCTCTTCCAGTACGCT
